# Rice breeding for yield under drought has selected for longer flag leaves and lower stomatal density

**DOI:** 10.1093/jxb/erab160

**Published:** 2021-04-14

**Authors:** Santosh Kumar, Santosh Tripathi, Suresh Prasad Singh, Archana Prasad, Fahamida Akter, Md Abu Syed, Jyothi Badri, Sankar Prasad Das, Rudra Bhattarai, Mignon A Natividad, Marinell Quintana, Challa Venkateshwarlu, Anitha Raman, Shailesh Yadav, Shravan K Singh, Padmini Swain, A Anandan, Ram Baran Yadaw, Nimai P Mandal, S B Verulkar, Arvind Kumar, Amelia Henry

**Affiliations:** 1 ICAR Research Complex for Eastern Region, Patna, Bihar, India; 2 Nepal Agricultural Research Council Regional Agriculture Research Station, Nepalgunj, Khajura, Banke, Nepal; 3 Regional Agricultural Research Station, Tarahara, Sunsari, Nepal; 4 Bihar Agricultural University, Sabour, Bihar, India; 5 Indira Gandhi Agricultural University, Raipur, Chhattisgarh, India; 6 Bangladesh Rice Research Institute, Regional Station, Rajshahi, Bangladesh; 7 ICAR Indian Institute of Rice Research, Rajendranagar, Hyderabad, Telangana, India; 8 ICAR Research Complex for North Eastern Hill Region, Lembucherra, Tripura, India; 9 International Rice Research Institute, Los Baños, Laguna, Philippines; 10 International Rice Research Institute South Asia Hub, ICRISAT, Patancheru, Telangana, India; 11 Banaras Hindu University, Varanasi, Uttar Pradesh, India; 12 ICAR National Rice Research Institute, Cuttack, Odisha, India; 13 National Rice Research Program, Hardinath, Baniniya, Janakpurdham, Nepal; 14 Central Rainfed Upland Rice Research Station, Hazaribag, Jharkand, India; 15 CSIRO Agriculture and Food, Australia

**Keywords:** Breeding, drought, flag leaf, G×E, *Oryza sativa*, physiology, rice, stomatal density, vapor pressure deficit

## Abstract

Direct selection for yield under drought has resulted in the release of a number of drought-tolerant rice varieties across Asia. In this study, we characterized the physiological traits that have been affected by this strategy in breeding trials across sites in Bangladesh, India, and Nepal. Drought- breeding lines and drought-tolerant varieties showed consistently longer flag leaves and lower stomatal density than our drought-susceptible check variety, IR64. The influence of environmental parameters other than drought treatments on leaf traits was evidenced by close grouping of treatments within a site. Flag-leaf length and width appeared to be regulated by different environmental parameters. In separate trials in the Philippines, the same breeding lines studied in South Asia showed that canopy temperature under drought and harvest index across treatments were most correlated with grain yield. Both atmospheric and soil stress strengthened the relationships between leaf traits and yield. The stable expression of leaf traits among genotypes and the identification of the environmental conditions in which they contribute to yield, as well as the observation that some breeding lines showed longer time to flowering and higher canopy temperature than IR64, suggest that selection for additional physiological traits may result in further improvements of this breeding pool.

## Teaser

Characterization of physiological traits affected by rice breeding for grain yield under drought conditions shows that atmospheric and soil stresses strengthen the relationship between leaf traits and yield across extensive trials in South Asia.

## Introduction

In order to decide which plant traits should be selected for in crop improvement, it is necessary to understand the state of traits in the current breeding pool (i.e. varieties currently available to farmers or breeding lines recently developed by breeders). The targeting of plant traits for crop improvement can also be guided by characterizing how plant traits relate to genotype × environment effects, since the contribution of different plant traits to grain yield may vary in different environments.

The breeding program for drought tolerance at the International Rice Research Institute (IRRI) has followed a strategy of direct selection for yield under drought (Kumar *et al.*, 2014). Following development of rice breeding lines and screening for yield under drought and well-watered conditions at IRRI, the process has included annual multi-location testing of lines in a breeding network across drought-prone rice-growing regions under both well-watered and drought conditions in ‘Advanced Yield Trials’ (AYT) followed by evaluation of selected lines by regional scientists and farmers in ‘Participatory Varietal Selection’ (PVS) trials. This drought-breeding pipeline has resulted in an annual genetic gain of 0.89%–1.9% (under moderate–severe drought stress; Kumar *et al.*, 2021) and the release of a number of drought-tolerant varieties that are now being cultivated by farmers in drought-prone areas, such as in Bangladesh, India, and Nepal (Supplementary Table S1). This simultaneous evaluation of the same genotypes across many locations also provides an excellent opportunity to investigate how direct selection for yield, as well as environmental variation across the drought-breeding network sites, has affected individual plant traits.

Detailed physiological characterization has been reported for two of the recently-released drought-tolerant rice varieties from IRRI: Sahbhagi dhan (as named in India; also named ‘BRRI dhan 56’ in Bangladesh and ‘Sukha dhan 3’ in Nepal) has shown a high rate of emergence under dry soil conditions, lateral root plasticity, and high harvest index under drought compared with check varieties and other breeding lines (Anantha *et al.*, 2016), and IR64 Dtr1 (DRR dhan 42) has exhibited high root hydraulic conductance, low canopy temperature, and greater root length density at depth under drought (Swamy *et al.*, 2013; Henry *et al.*, 2015). These results have highlighted the importance of multiple plant traits for conferring improved rice yield under drought that is stable across test sites, and that different combinations of traits can result in improved yield in individual drought-tolerant genotypes.

In addition to the varying relationships between individual plant traits and grain yield (depending on the traits already present in a given genetic background), environmental parameters may have varying effects on different genotypes. Given the large range of environmental characteristics among drought-prone, rice-growing regions (e.g. Inthavong *et al.*, 2011; Singh *et al.*, 2017; Tanaka *et al.*, 2017), it is important to understand how the traits respond to environmental variation if they are to be targeted for selection in crop improvement. Factors affecting soil moisture (e.g. soil type, rainfall) are perhaps the most obvious in causing variability in the relationships between plant traits and grain yield in drought-prone regions, and recently-developed rice breeding lines have shown a large degree of plasticity to soil moisture levels in terms of shoot development, flowering time, and root growth (Henry *et al.*, 2015; Vikram *et al.*, 2015; Anantha *et al.*, 2016; Sandhu *et al.*, 2016; Grondin *et al.*, 2018). However, atmospheric conditions in drought-prone environments can also have a strong effect on plant traits, especially leaf development (e.g. Devi *et al.*, 2015; Devi and Reddy, 2018; Choudhary *et al.*, 2020). Although the variety Sahbhagi dhan typically shows high and stable yield under drought across sites (Kumar *et al.*, 2012), yields have been lower than expected in environments with low early-season temperatures or with low solar radiation, and this was related to reduced shoot biomass (Anantha *et al.*, 2016). Another example is the response to vapor pressure deficit (VPD) and light levels; recently-developed upland rice breeding lines were better able to adjust their stomatal conductance in response to light and VPD than check varieties (Henry *et al.*, 2019). These examples point to the importance of characterizing atmospheric conditions in addition to soil moisture when assessing plant traits related to grain yield across environments.

In this study, we made use of the ongoing rice breeding AYT–PVS pipeline to observe how selection for grain yield over time has affected certain plant traits. In multi-location trials in South Asia, we focused on measuring flag-leaf dimensions and stomatal density together with environmental parameters hypothesized to be affecting plant traits. In trials at IRRI in the Philippines, we conducted additional measurements to further dissect the plant traits that have been affected by direct selection for yield under drought. By improving our understanding of the current pool of drought-tolerant rice breeding lines and varieties (both in terms of plant traits and environmental response), our aim was to identify targets for further improvement of drought tolerance in rice.

## Materials and methods

To assess the effects of selection for yield under drought on physiological traits in rice (*Oryza sativa*), we characterized three sets of advanced drought-breeding lines and drought-tolerant varieties across two groups of environments: (1) South Asia trials at 12 varietal testing sites in drought-prone, rice-growing regions of Bangladesh, India, and Nepal (Supplementary Table S2), in which a limited number of measurements were conducted; and (2) IRRI trials in the Philippines (14.1667N, 121.25E), in which more detailed characterization was conducted.

### Genetic material

We selected subsets of entries in ongoing multi-location trials of larger sets that were undergoing evaluation for varietal release (Supplementary Table S1). All lines were developed in the IRRI drought-breeding program as described by Kumar *et al.* (2014). The same sets of genotypes measured in the multi-location trials in South Asia were grown in the detailed characterization experiments at IRRI. Nine ‘Participatory Varietal Selection’ (PVS) lines were measured (Set 1: 2016 in South Asia, and 2016 wet season and 2017 dry season at IRRI). Subsets of 12 ‘Advanced Yield Trials’ (AYT) lines and checks were chosen based on their inclusion in the greatest number of trials across the three South Asian countries and designated as Set 2 in 2017 in South Asia and 2017 wet season–2018 dry season at IRRI, and Set 3 in 2018 in South Asia and 2018 wet season–2019 dry season at IRRI. In addition to the test entries, the variety IR64 was considered as the check in all trials, except in the 2017 (Set 1) South Asia trials in which Sahbhagi dhan was used since IR64 was not planted in the PVS trials. IR64 is a common check variety that was at one time grown on millions of hectares in Asia, and it has been characterized as sensitive to drought at the reproductive stage (Mackill and Khush, 2018). IR64 also forms the genetic background for some of the drought-tolerant lines in this study. The remaining entries measured in PVS trials were advanced drought-breeding lines, and the remaining entries in AYT trials were a combination of advanced drought-breeding lines and released varieties planted as checks in those trials but treated as test entries in this study.

### South Asia trials

All South Asia trials were conducted in the ‘Aman’/‘kharif’/‘Barkha’ (wet) seasons except for the Cuttack drought-treatment trials, which were conducted in the ‘rabi’ (dry) season. The PVS trials were conducted under irrigated control (CONT) conditions at five sites, and under reproductive-stage drought stress (REPST) conditions at three sites, whilst the AYT trials included both CONT and REPST conditions at all sites (12 sites for Set 2 and 11 sites for Set 3). The experiments were laid out in a randomized complete block design for PVS trials and an alpha-lattice design for AYT trials, both with three replications. At some AYT trial sites, sowing of the REPST trial was delayed by 2–3 weeks compared to the CONT trial, so that the reproductive stage of the crop was synchronized with a higher probability of drought due to the withdrawal of the monsoon. At Raipur, an additional rainfed direct seeded trial was also included.

Individual rice seedlings at 21–25 d old were manually transplanted into 8-m^2^ (PVS) and 4-m^2^ (AYT) plots in puddled fields at a spacing of 15 cm within a row and 20 cm between rows. Raipur direct-seeded rice (DSR) trials were directly sown into the experimental field in rows spaced at 20 cm on a leveled and tilled sandy loam soil at a seed rate of 8 g m^–2^ in 2.8-m^2^ plots. In the CONT trials, nitrogen was applied in three equal measures as a basal application, and at the maximum tillering and panicle initiation stages, while P_2_O_5_ and K_2_O were applied as basal fertilizer. In the REPST trials, nitrogen was applied in two equal measures as a basal application and at the maximum tillering stage (before water was drained to create stress) while P_2_O_5_ and K_2_O were also applied as basal fertilizer. In both treatments of the PVS and AYT trials, the total ratio of N:P:K fertilizer applied was 90:50:50. Weeds were controlled in all South Asia lowland trials using a combination of post-emergence herbicide (Nominee Gold: bispyribacsodium 0.025 kg a.i. ha^–1^) and hand weeding. In the Raipur DSR trials, one pre-emergence herbicide (Butachlor) and one post emergence herbicide (Nominee Gold) were used to control weeds.

Standing water was maintained in the CONT trials from transplanting up to 20 d before maturity by rainfall and/or by providing supplementary irrigation as required. In the REPST trials, standing water was maintained up to 50–60 d after sowing, after which the field was drained to allow it to dry for stress development. No supplemental irrigation was provided after drainage until harvest. No irrigation was applied in the Raipur DSR trials as they were completely rainfed.

### IRRI trials

Each set of genotypes was grown in one wet season (WS) and one dry season DS) at IRRI, with well-watered control (CONT) and reproductive-stage drought (REPST) treatments applied each season. Each genotype was transplanted (3–5 seedlings per hill) at ~17 d after sowing (DAS) in four replicates in a randomized complete block design in 3-m^2^ plots with 25-cm spacing between rows and 20-cm spacing within rows. Unlike the South Asia trials from which a subset of lines were selected to measure, for this study the test entries and the IR64 check were the only genotypes planted in each of the IRRI trials. Each treatment received a ratio of 50:50:50 N, P, and K as basal fertilizer at the time of transplanting and 50 kg ha^–1^ (NH_4_)_2_SO_4_ as topdressing at least 21 d after transplanting. Weeds were controlled by hand weeding, and snails were controlled using a molluscicide (Baluscicide: niclosamide 0.008 kg a.i. ha^–1^). Drought-stress treatments in the IRRI trials were initiated between 41–53 DAS by withholding irrigation. The drought treatments in 2018DS, 2018WS, and 2019DS were applied by growing the plants in an automated rainout shelter, but they were rewatered once or twice during the season (Supplementary Table S3) to ensure harvestable grain yields.

### Environmental characterization

Soil and climate data were collected across sites to assess how the measured physiological traits responded to environmental conditions. A survey of soil characteristics across the South Asia trial sites (Singh *et al.*, 2017) was incorporated into the environmental characterization in this study. In addition, soil water status was monitored in many of the South Asia trials and in all IRRI trials using perforated water-table tubes to a depth of 1 m together with tensiometers (Soilmoisture Equipment Corp., Goleta, CA, USA) installed at a depth of 30 cm. At sites where weather stations were available, rainfall during the drought-stress treatment (60–100 DAS) together with temperature and relative humidity (RH) were collected from 0–120 DAS. Having obtained initial results indicating potential relationships between leaf dimensions and atmospheric conditions in 2017, we were then able to procure weather-station data for an increased number of South Asia sites in the 2018 trials.

We focused on vapor pressure deficit (VPD) as a key parameter to characterize differences in atmospheric conditions across sites, with the aim of determining the mean maximum daily VPD per season. When available, we used the maximum daily temperature together with the RH in the afternoon (14.00–15.00 h) to calculate the VPD in both the South Asia and IRRI trials. In cases where maximum values were not available, the VPD was calculated from the mean daily temperature (*T*) and humidity as [(100–RH)/100]×0.6569*e*^0.0619*T*^.

### Physiological and agronomic measurements

The measurements conducted in the South Asia trials were chosen on the basis of being practical to perform during the timeframe of a tour across the sites, whilst the measurements at IRRI were chosen in order to provide additional insights into the performance to the drought-breeding lines within a longer timeframe for sample processing.

Flag-leaf dimensions (length and maximum width) were determined from three randomly selected leaves per plot in 2–3 replicates of the South Asia trials (~90–105 DAS) and from all four replicates at IRRI (69–104 DAS). In the South Asia trials, the flag leaves in Set 1 were imaged alongside a standard size scale on a white background and measured using ImageJ v 1.49b (Abràmoff *et al.*, 2004), whilst for Sets 2 and 3 they were measured manually. In the IRRI trials, the dimensions and areas of 2–3 flag leaves per plot were determined in all replicates using a roller-belt leaf area meter (LI-COR LI-3100). The samples from the IRRI trials were subsequently dried and weighed, and specific leaf area was calculated (area divided by dry weight).

Stomatal density was determined for the penultimate leaf on the plant, using two fully expanded leaves per plot that were selected randomly from two replicates in Set 2 of the South Asia trials (60–114 DAS) and four replicates in all IRRI trials (55–71 DAS). In order to obtain stomatal imprints, a thin layer of transparent nail polish was applied to an area of ~5 cm^2^ of the adaxial side of the leaf and allowed to dry before being peeled off using transparent adhesive tape, according to (Kusumi, 2017). In all South Asia trials and in the 2016WS and 2017DS IRRI trials, stomatal imprints were collected directly from the field. In the 2017WS–2019DS IRRI trials, in order to improve the quality of the imprints, leaf samples were collected from the field and placed in 50-ml open-topped Falcon tubes filled with water and brought to the laboratory before the nail polish was applied.

Microscopic observation of the stomatal imprints from all trials was conducted at IRRI. Each imprint was placed on a glass slide and observed at 20× magnification using either a Zeiss Axioplan 2 with an Olympus DP70 imaging system or an Olympus BX51 and DP71 imaging system. Three representative areas per imprint were imaged and analysed using ImageJ v.1.49b. Since all stomatal density measurements were conducted within individual interveinal areas, in the South Asia Set 2 trials we also compared the area measured, interveinal distance, number of rows of stomates, and the height and width of individual stomates in order to investigate their contributions to the observed trends in stomatal density. In total, ~1600 images and >20 000 individual stomata were measured.

The sap bleeding rate from the root zone was measured at 64–77 DAS in all IRRI trials on one hill per plot according to the methods described by (Morita and Abe (2002) and Henry *et al.* (2012). The shoots of an entire hill were cut at a height of ~15 cm from the soil surface, wrapped in a pre-weighed cotton towel covered with plastic, the exuded sap was collected for 4 h starting ~2.5 h after dawn, and then the towel and plastic together with the collected sap was re-weighed. The sap bleeding rate was calculated as the difference between the weights divided by the dry weight of the shoots. The top angle of the root crown was determined at 50–69 DAS in all the IRRI drought trials except in 2018WS on one root crown per plot. The root crowns were excavated using a shovel at a radius of ~10 cm around each hill and to a depth of ~25 cm. The root crowns were washed, imaged alongside a size standard, and ImageJ was used to determine the angle from horizontal at the root–shoot junction. Only the drought treatment was considered for this analysis since the nodal roots appeared more rigid than those in the well-watered treatments and were therefore more likely to retain their angle following excavation.

Canopy temperature in the IRRI trial drought-stress treatments was measured across five dates in 2018DS, 14 dates in 2018WS, and 18 dates in 2019DS using three infrared sensors (Apogee Instruments, Logan, UT, USA) angled at 45° and mounted at a height of 1 m above the soil surface on a semi-automated instrument rack, aligned with the planted rows, that rolled along the rails of the rainout shelter. Each sensor collected data along the length of the row, and the measurements were averaged to obtain one canopy temperature reading per plot per measurement date. The dates were then averaged to obtain one mean canopy temperature value per plot per season. IRRI trials conducted outside the rainout shelter were not subject to canopy temperature measurements (2016WS–2017WS drought trials and all well-watered trials).

In the South Asia trials, plots were harvested at maturity and the threshed grain was dried to determine yields at 12 % moisture content. Agronomic traits determined in the IRRI trials were as follows: plant height (from three hills per plot during the reproductive stage), days to 50% flowering, grain yield (normalized to 14% grain moisture content), and straw biomass at harvest (determined from a 1.5 m^2^-area), and harvest index.

### Statistical analysis

Since the flag-leaf and stomatal density data collected in the South Asia trials were from subsets of larger experiments in an alpha lattice design, we analysed these subsets as randomized complete block designs for each trial. We used Type III ANOVA with Satterthwaite’s method for testing genotypic effects in these trials and the –2 log likelihood ratio to test for the significance of environment effect. The library packages used in R v.3.6.2 (www.r-project.org) were lme4, lmerTest, agricolae, and lsmeans for the ANOVA and *post hoc* results. The R function prcomp was used to conduct principal component analysis on flag-leaf length and width together with a range of environmental parameters characterized at each experimental site in South Asia in 2017–2018. We conducted imputation using the missMDA package to fill in some missing data, namely for 20% of the tensiometer data and 9.3% of the water table data. A selection of sites with the most complete environmental data was included to empower the validity of the imputation method. Cluster analysis was also used to display the grouping effects for the South Asia sites. Principal component analysis for the IRRI trials was also conducted using prcomp but without data imputation. Statistical Tool for Agricultural Research (STAR, v.2.0.1; http://bbi.irri.org/products) was used to analyse the genotypic effects on physiological traits for each season of the IRRI trials. Pearson correlation was applied to traits for both the South Asia and IRRI trials.

## Results

### Variation in environmental conditions across the trial sites

Among sites in South Asia, Rajshahi and Tripura stood out for having high RH and low VPD, whilst the Cuttack REPST trials stood out for having low RH and high VPD (~2.5 kPa; Supplementary Fig. S1A, B). Hazaribag exhibited some of the lowest mean temperatures (~18 °C) and Nepalgunj the highest (~36 °C; Supplementary Fig. S1C, D). Soil water status and rainfall amounts were variable across sites and years, and were less frequently recorded than the other environmental parameters (Supplementary Fig. S1 E–I). Patna and Hazaribag showed the lowest rainfall during the reproductive stage of the trials in both 2017 and 2018 (as low as 36 mm).

Compared to the trials in South Asia, less variation was observed in RH and VPD among seasons at IRRI (Supplementary Table S3). In general, plants grown in 2017WS–2018DS at IRRI in Set 2 experienced the least severe drought stress, with mean soil water potential values at 30 cm depth of –1.3 to –10.3 kPa.

### South Asia trials: genotype and environment differentially affected flag-leaf length and width

**Fig. 1.**
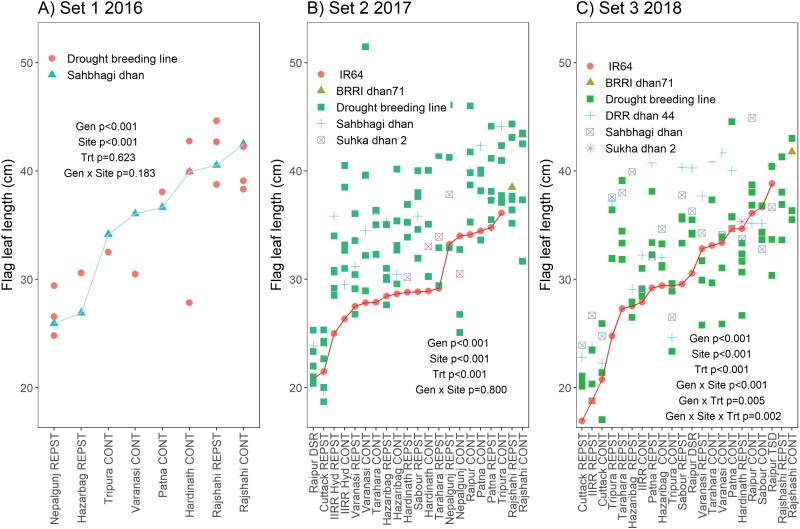
Flag-leaf length of rice across experimental sites and drought treatments in South Asia in the three sets of drought-breeding lines. The sites on the *x*-axes are arranged in order of the mean flag-leaf length of the check varieties Sahbhagi dhan in 2016 and IR64 in 2017 and 2018 (highlighted by lines), except for Rajshahi as IR64 was not grown at that site. *P*-values are shown for genotype (Gen), site, drought treatment (Trt), and genotype × site, plus any other significant interactions with genotype, across all experiments grown for each Set. Sites are listed in Supplementary Table S2 and the genotypes in each set are shown in Supplementary Table S1. CONT, control (well-watered); REPST, drought stress during the reproductive period.

**Fig. 2.**
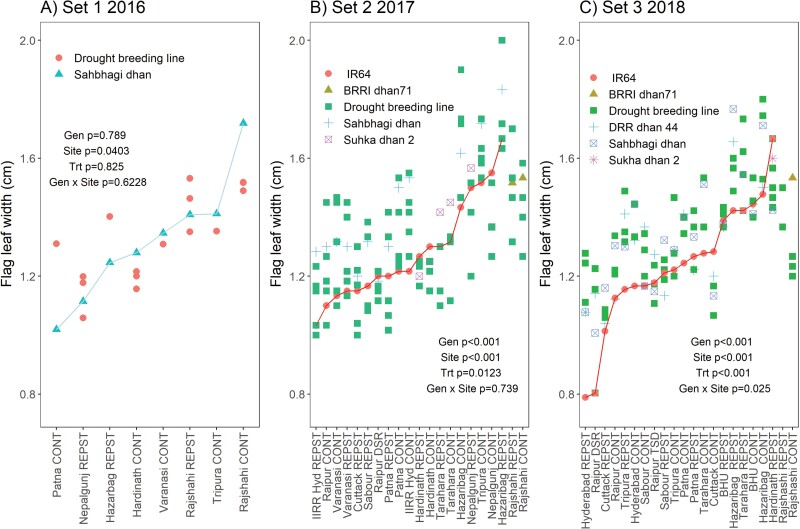
Flag-leaf width of rice across experimental sites and drought treatments in South Asia in the three sets of drought-breeding lines. The sites on the *x*-axes are arranged in order of the mean flag-leaf width of the check varieties Sahbhagi dhan in 2016 and IR64 in 2017 and 2018 (highlighted by lines), except for Rajshahi as IR64 was not grown at that site. *P*-values are for genotype (Gen), site, drought treatment (Trt), and genotype × site across all experiments grown for each set. Sites are listed in Supplementary Table S2 and the genotypes in each set are shown in Supplementary Table S1. CONT, control (well-watered); REPST, drought stress during the reproductive period.

Flag leaves were consistently longer in the drought-breeding lines and released varieties across sites and seasons in South Asia compared with the drought-susceptible check variety IR64 (Fig. 1, Supplementary Table S4). In addition to the genotype effects, site and drought treatment showed significant effects on flag-leaf length, except in 2016 when the drought effect was not significant (Fig. 1). The genotypic differences were more distinct in Set 2 grown in 2017 (Supplementary Table S4). Genotypic differences in flag-leaf width were not as consistent as observed for length (Fig. 2, Supplementary Table S4). Within each site, the drought and control treatments tended to show similar flag-leaf length and width, except in cases of very severe drought such as Raipur DSR in 2017. Among sites, flag-leaf length was consistently greatest in both treatments at Rajshahi across seasons (Fig. 1).

**Fig. 3.**
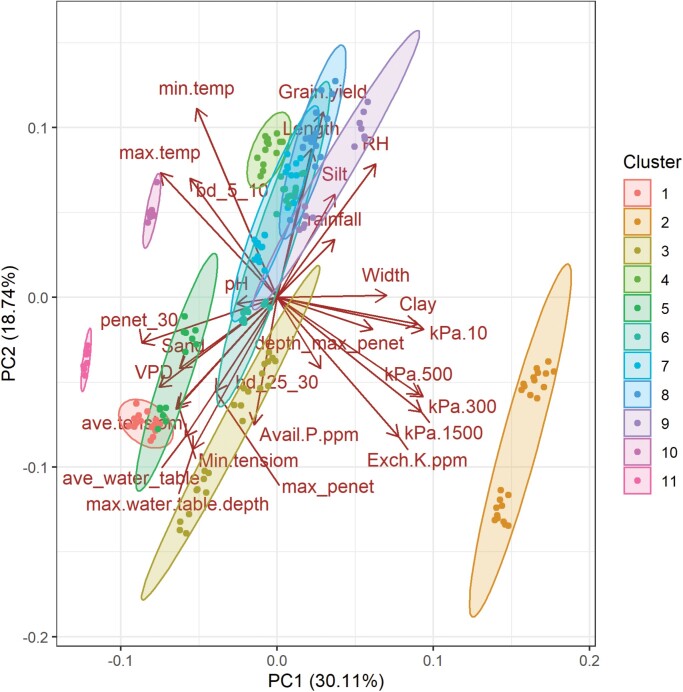
Principal component analysis of rice flag-leaf length and width together with a range of environmental parameters characterized across 31 trials at nine sites in South Asia in 2017–2018. A total of 22 genotypes were considered (Supplementary Table S1). The names of the variables are defined in Table 1, and the trials and genotypes associated with each cluster are given in Supplementary Table S6. The circles represent genotypes in each trial, which largely clustered together by site over the two seasons considered., The shaded areas represent the different clusters.

To better understand the site and treatment effects on flag-leaf and width, we ran a principal component analysis (PCA) on the data together with a range of environmental parameters characterized during each trial, and including soil characterization previously reported from the South Asia sites (Singh *et al.*, 2017). Among the environmental parameters, soil clay content and values derived from water retention curves showed the highest loading values in PC1, whilst the minimum temperature, mean and maximum water-table depths, and soil P and K levels showed the highest loading values in PC2 (Supplementary Table S5). Among the sites, Hazaribag showed the highest loading values in PC1, and the Sabour REPST treatment showed the highest loading values in PC2 (Supplementary Table S6). Genotypes did not group for loading values. Both years and treatments within an individual site clustered together, except for Patna and Varanasi where the treatments fell in separate clusters. The only two sites that clustered together were Cuttack CONT with the Raipur CONT and REPST treatments (Fig. 3, Supplementary Table S6).

Many environmental parameters in the South Asia trials were directly correlated with both flag-leaf length and width; however, rainfall, bulk density at 25–30 cm, soil pH, available P, % sand and silt, and maximum penetrometer reading were only correlated with length, whereas maximum temperature, minimum tensiometer reading, exchangeable K, % clay, and water retention from 10–500 kPa were only correlated with width (Table 1). Parameters showing significant but opposite relationships with flag-leaf length and leaf width were minimum temperature and bulk density at 5–10 cm. In the PCA bi-plot, flag-leaf length grouped with positive effects on rainfall, RH, and % silt, and with negative effects on soil P and with the mean and extreme values of water-table depth and tensiometer readings (Fig. 3). Flag-leaf width grouped with positive effects on the depth of the maximum penetrometer reading, % clay, soil water retention, bulk density at 25–30 cm, and soil K.

**Table 1.** Environmental parameters and their relationships with flag-leaf length and width as determined by Pearson’s correlation across 31 trials at nine sites in South Asia in 2017–2018

Parameter	Abbreviation	Pearson’s *r*	
		Length	Width
Flag-leaf length	Length	1.000	0.2652***
Flag-leaf width	Width	0.2652***	1.000
Relative humidity	RH	0.5292***	0.4829***
Vapor pressure deficit	VPD	–0.3978***	–0.496***
Max. daily temperature	max.temp	–0.072	–0.3344***
Min. daily temperature	min.temp	0.2732***	–0.3956***
Min. tensiometer reading 60–100 DAS	Min.tensiom	–0.3146***	–0.4034***
Average tensiometer reading 60–100 DAS	ave.tensiom	–0.4009***	–0.4542***
Max. water table depth 60–100 DAS	max.water.table.depth	–0.3918***	–0.2373**
Average water table depth 60–100 DAS	ave_water_table	–0.2842***	–0.278***
Rainfall 60–100 DAS	rainfall	–0.060	0.119
Bulk density 5–10 cm	bd_5_10	0.2**	–0.1934***
Bulk density 25–30 cm	bd_25_30	–0.3619***	0.122
pH	pH	0.2727***	–0.003
Available phosphorus	Avail.P.ppm	–0.095	–0.053
Exchangeable potassium	Exch.K.ppm	–0.064	0.4185***
% clay	Clay	–0.056	0.2301**
% silt	Silt	0.3774***	–0.012
% sand	Sand	–0.2751***	–0.077
Water retention at 10 kPa	kPa.10	–0.013	0.5683***
Water retention at 300 kPa	kPa.300	–0.100	0.4604***
Water retention at 500 kPa	kPa.500	–0.088	0.4089***
Water retention at 1500 kPa	kPa.1500	–0.141	0.4461***
Max. penetrometer reading	max_penet	–0.1688*	–0.061
Depth of max. penetrometer reading	depth_max_penet	0.1855*	0.3524***
Penetrometer reading at 30 cm depth	penet_30	–0.3371***	–0.4077***

DAS, days after sowing. *P<0.05; **P<0.01; ***P<0.001. A total of 22 genotypes were considered (Supplementary Table S1).

### South Asia trials: stomatal density was consistently lower in drought-breeding lines and released varieties than in IR64

**Fig. 4.**
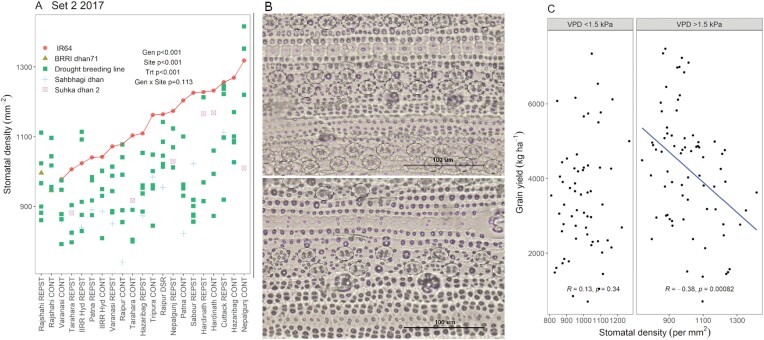
Stomatal density of the penultimate leaf on rice plants in the South Asia trials in Set 2 (2017). (A) Stomatal density across experimental sites and drought treatments. The sites on the *x*-axes are arranged in order of the mean value for the control variety IR64 (highlighted by the line), except for Rajshahi as IR64 was not grown at that site. *P*-values are shown for genotype (Gen), site, drought treatment (Trt), and genotype × site across all experiments. (B) Representative epidermal peels from the Cuttack site showing higher stomatal density in IR64 (top) compared to breeding line IR 98976-20-1-2-2 (bottom); circles indicate the stomata. (C) Effect of vapor pressure deficit (VPD) on the relationship between stomatal density and grain yield (additional parameters affecting this relationship are shown in Supplementary Fig. S2). Each point represents one genotype per trial, and the correlation analysis included 33 trials across 12 sites, with 28 genotypes considered. Sites are listed in Supplementary Table S2 and the genotypes in each set are shown in Supplementary Table S1. CONT, control (well-watered); REPST, drought stress during the reproductive period.

The stomatal density of the penultimate leaf measured across South Asia sites in 2017 was significantly affected by site and genotype, but not by drought treatment (Table 2, Fig. 4). The drought-breeding lines and released varieties showed consistently lower stomatal densities compared with the drought-susceptible check IR64. Stomatal density was negatively correlated with the area of leaf measured (which was within one interveinal region) and the number of stomatal rows per interveinal region, and the area measured increased with the number of rows per interveinal region (*r*=0.84; *P*<0.001; Table 2), but was not correlated with interveinal distance. Of all stomatal traits measured, only stomate width was affected by the drought treatment across sites (Table 2).

**Table 2.** Effects of drought treatment and genotype on stomatal density measurements, and correlations with leaf and stomatal traits measured in 21 trials across 12 South Asia sites in Set 2 (2017)

	Site effect	Treatment effect	Genotype effect	Site × Treatment	Correlation with stomatal density (*r*^2^)
Stomatal density	<0.001***	<0.001***	<0.001***	<0.001***	–
Interveinal area measured	<0.001***	0.745	0.768	0.254	–0.43***
Interveinal distance	<0.001***	0.166	0.008**	<0.001***	–0.003
No. rows per interveinal area	<0.001***	0.661	0.604	0.026*	–0.26***
Stomate width^a^	0.994	0.009**	0.796	1	–0.01
Stomate length	0.508	0.727	0.591	0.783	–0.04

^a^ Treatment × Genotype: *P*<0.001. *P<0.05; **P<0.01; ***P<0.001.

A total of 11 genotypes were considered (Supplementary Table S1).

### South Asia trials: atmospheric conditions, soil conditions, and genotype sets affected the relationships between leaf traits and grain yield

Across the South Asia trials, flag-leaf length showed a positive relationship with grain yield in Sets 2 and 3 (*n*>130; Supplementary Table S7) but not in Set 1 (*n*=28), which agreed with the close grouping of leaf length and grain yield across Sets 2 and 3 (Fig. 3). Stomatal density in Set 2 showed a negative relationship with grain yield (Supplementary Table S7), and flag-leaf width showed a positive relationship with grain yield in Set 2 only. However, the relationships between leaf traits and grain yield changed when the trials were grouped by environmental conditions, and these changes sometimes differed between the different sets of genotypes (Fig. 4, Supplementary Fig. S2). Notably, the relationship between stomatal density and grain yield was negative in trials with high VPD (>1.5 kPa) but no relationship was observed in trials with low VPD (Fig. 4C). A similar trend of stomatal density negatively correlating with grain yield was observed in trials with high maximum temperature (>32 °C), low rainfall (<170 mm), high soil penetrometer readings (>2500 kPa), and low clay content (<26%; Supplementary Fig. S2). The conditions that eliminated the positive relationship between flag-leaf length and grain yield (low VPD, high rainfall, and low maximum temperature) were observed only in Set 3; the relationship was maintained across conditions in Set 2 (Supplementary Fig. S3). In contrast, both Sets 2 and 3 showed a loss of the positive relationship between flag-leaf width and grain yield under low maximum temperature, high rainfall, and low soil penetrometer readings, and only Set 2 was sensitive to soil clay content (Supplementary Fig. S4).

### IRRI trials: canopy temperature and harvest index were most closely related to grain yield

Similar to the South Asia trials, the drought-breeding lines and released varieties typically showed longer flag-leaf length and lower stomatal density compared to IR64 in the IRRI trials (Supplementary Fig. S5, Supplementary Table S8). The genotypic differences in flag-leaf width were inconsistent in comparison with IR64, as observed in the South Asia trials. All dry season trials resulted in narrower leaf width than all wet season trials across the three sets of genotypes grown at IRRI (Supplementary Fig. S5). The specific leaf area of flag-leaves at IRRI was lower in the drought-breeding lines and released varieties compared with IR64 only in Set 1 (2016WS–2017DS). Among the root traits measured, the root crown top angle of IR64 varied differently in comparison with the drought-breeding lines and released varieties, and it was not consistent among genotype sets (Supplementary Fig. S6, Supplementary Table S8). The sap bleeding rate was generally lower under drought compared to the well-watered treatments, but also did not show consistent genotype or season effects.

**Fig. 5.**
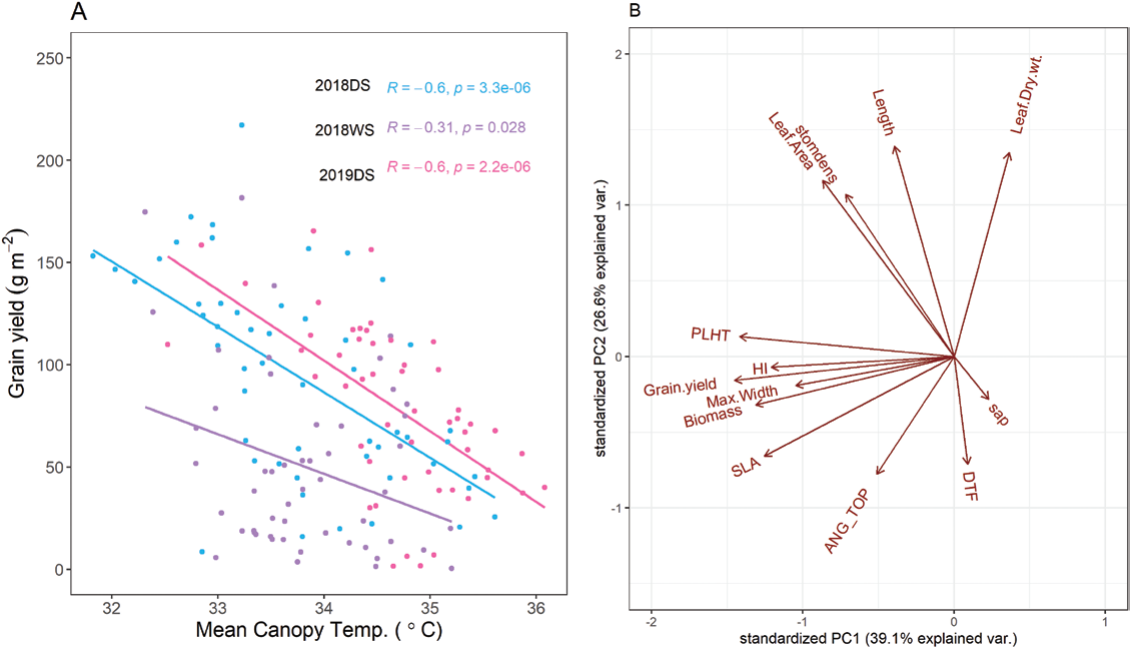
Effect of canopy temperature on grain yield in rice and principal component analysis of traits in the IRRI trials. (A) Linear regressions between grain yield and mean canopy temperature across five, 14, and 18 measurement dates in 2018DS, 2018WS, and 2019DS, respectively. Each point represents one experimental plot, and genotypes are listed in Supplementary Table S2. DS, dry season; WS, wet season. (B) Principal component analysis of leaf, root, and agronomic traits measured across all IRRI trials. Mean canopy temperature was only measured in the 2018DS–2019DS stress treatments and is not included. Length, flag-leaf length; Leaf.Dry.wt: flag-leaf dry weight; sap, xylem sap bleeding rate; DTF, days to flowering; ANG_TOP, root crown top angle; SLA, specific leaf area of the flag leaf; Biomass, straw biomass at harvest; Max.Width, maximum flag-leaf width; Grain.yield, grain yield at harvest; HI, harvest index; PLHT, plant height at flowering; Leaf.Area, flag-leaf area; stomdens, stomatal density of the penultimate leaf on the plant.

In terms of agronomic traits, almost all the drought-breeding lines and released varieties showed a longer time to flowering than IR64 except Sahbhagi dhan (Supplementary Fig. S7, Supplementary Table S8). While the genotype, season, and drought effects on biomass were not consistent, overall IR64 showed the lowest grain yield and harvest index across the trials. To better understand the specific physiological traits that were most related to grain yield, we ran correlation analysis and a PCA across the IRRI trials. Harvest index was significantly correlated with grain yield in 11 out of 12 trials (drought and well-watered treatments) (Table 3). The leaf and root traits measured generally did not show consistent direct relationships with grain yield within individual trials, but canopy temperature was negatively correlated with grain yield in all three trials (Fig. 5A, Table 3). Genotypic variation for canopy temperature indicated that IR64 did not stand out for having extreme values compared to the drought-breeding lines and released varieties (Supplementary Table S8). In the PCA across all the IRRI trials, flag-leaf width grouped together with grain yield, whilst leaf length did not (Fig. 5B). Unlike in the South Asia trials, no cut-off values for environmental parameters affecting the relationships between leaf traits and grain yield could be identified from the more limited number of IRRI trials.

**Table 3.** Correlations of physiological traits with grain yield in the IRRI trials

Trait	Correlation with grain yield (Pearson’s *r*)					
	Set 1		Set 2		Set 3	
	2016WS	2017DS	2017WS	2018DS	2018WS	2019DS
Drought stress treatments						
Root crown top angle	0.0555	–0.1702	–0.0356	–0.0263	–	–0.0855
Sap bleeding rate	–0.1319	0.3325*	0.1543	0.1857	–0.0923	–0.0211
Biomass	0.315	0.0853	–0.1453	0.404**	0.6444***	0.3188*
HI	0.9257***	0.9569***	0.8677***	0.941***	0.9573***	0.955***
PLHT	0.2272	0.6482***	0.3168*	0.4099**	0.5815***	0.3673**
Stomatal density	–0.0653	–0.4849	0.2461	0.1112	0.0506	–0.0568
Flag-leaf area	0.304	0.6011	0.2251	0.5068***	0.2673	–0.2101
Flag-leaf width	0.0378	0.2413	0.1873	0.4334**	0.1436	–0.2288
Flag-leaf length	0.2813	0.6186***	0.1595	0.2854*	0.162	–0.0665
Flag-leaf dry weight	0.3445	0.575***	0.2174	0.4855***	0.2481	–0.1379
Flag-leaf SLA	–0.337	0.094	0.0055	–0.0378	–0.0394	0.1813
DTF	0.5711***	–0.0761	0.1236	–0.1347	–0.3855**	–0.0555
CT^a^				–0.5952***	–0.3054*	–0.6031***
Well-watered treatments						
Sap bleeding rate	–0.3826*	0.1251	–0.0945	–0.1554	–0.1418	0.2247
Biomass	0.5449**	0.7298***	0.4528**	0.0394	0.6974***	0.6757***
HI	0.4739**	0.6157***	0.4955***	0.4035**	0.1234	0.5015***
PLHT	0.4772**	–0.2626	0.5732***	0.1591	0.2033	0.1018
Stomatal density	0.247	0.1824	–0.0914	–0.3457*	–0.0310	–0.1301
Flag-leaf area	0.197	0.0493	0.3214*	0.1034	0.2677	–0.1504
Flag-leaf width	0.1046	–0.1091	0.3061*	0.2873*	0.0542	–0.133
Flag-leaf length	0.1124	0.1725	0.2127	0.0172	0.2617	–0.1046
Flag-leaf dry weight	0.1571	0.3297*	0.2901	0.1097	0.2324	0.0022
Flag-leaf SLA	0.0841	–0.5548***	0.1899	0.0102	–0.0775	–0.1377
DTF	0.0882	0.3756*	0.3253*	0.2958*	–0.0672	0.4334**

^a^ Canopy temperature measurements were not made in trials conducted outside the rainout shelter (2016WS–2017WS drought trials and all well-watered trials). DS, dry season; WS wet season.

WS, wet season; DS, dry season; HI, harvest index; PLHT, plant height at flowering; SLA, specific leaf area; DTF, days to flowering; CT, canopy temperature. The genotypes in each set are shown in Supplementary Table S1. Genotype, season, and drought treatment effects for each trait measured are given Supplementary Tables S5–S7. *P<0.05; **P<0.01; ***P<0.001.

## Discussion

Although breeding for rainfed environments has historically reported large genotype × environment (G×E) effects on yield in rice (e.g. Basford and Cooper, 1998), the stability of the leaf traits that we observed in the drought-breeding lines and released varieties across rainfed environments in this study was notable. Similarly, we had previously observed that genetic differences in transpiration efficiency and lateral root development related to the major-effect drought-tolerance quantitative trait locus *qDTY*_*12.1*_ were more stable than genetic differences in grain yield across a range of sites and conditions (Henry *et al.*, 2014). This stability in physiological traits together with our identification of atmospheric and soil conditions under which those traits confer a yield advantage (Fig. 4, Supplementary Figs S2–S4), suggest a strong potential for the effectiveness of further improving yield across rainfed rice environments by selecting for physiological traits (Reynolds and Langridge, 2016). The key step in that process would be to identify what potentially beneficial traits are not already present in the breeding pool.

Larger flag leaves have long been hypothesized to be related to grain yield in grasses (e.g. Simpson, 1968), but the effect of drought on flag-leaf dimensions and their relationship with grain yield in rice is less well defined. In the current study, it was interesting that genotypic differences in flag-leaf length were more consistent than those in width, and length appeared to be more strongly selected for during the breeding process (as indicated by differences with IR64: Figs 1, 2, Supplementary Table S4), but width was just as frequently, if not more, related to grain yield (Fig. 5B, Supplementary Figs. S3–S4). Furthermore, it appeared that flag-leaf length was more affected by atmospheric conditions (rainfall and RH) whilst width was more affected by soil hydraulic properties (clay content and soil water retention; Fig. 3). Similarly, leaf length and width in maize have been reported to be differentially affected by environmental parameters, with elongation being negatively related to evaporative demand and widening being reduced by lower levels of intercepted light (Lacube *et al.*, 2017). It is not clear if light levels affected flag-leaf length or width in the current study, although we observed distinctly larger leaf width in IRRI in wet seasons (during which plants typically receive less solar radiation) than in dry seasons (Fig. 2), which suggests the opposite trend to that observed in maize.

Stomatal density of the penultimate leaf was also an interesting trait to examine in the drought-breeding lines and released varieties given the consistent ranking of IR64 across environments (Fig. 4A) and its clear negative relationship with yield in environments with high evapotranspiration and low soil moisture (Fig. 4C, Supplementary Fig. S2). Within an individual genotype, rice stomatal density has been shown to decrease under drought stress (e.g. Ouyang *et al.*, 2017), but the absence of significant G×E effects on stomatal density was notable in our current study: the values in drought-breeding lines and released varieties were lower than IR64 even in well-watered trials. However, reduced stomatal density may not necessarily result in reduced photosynthesis or stomatal conductance (i.e. yield-limiting traits in well-watered conditions), as reported by Caine *et al.* (2019) using *OsEPF1* mutants. Therefore, it is possible that stomatal aperture (or other traits affecting stomatal conductance that were not measured in the current study) might have shown greater G×E effects rather than stomatal density, which could also explain why the relationship between stomatal density and yield was only observed in environments with high evapotranspiration.

It should be noted that the field of view selected for the measurement of stomatal density has a strong effect on the values recorded. Our epidermal peels were sampled in many locations and transported long distances before analysis, and because of quality issues we used relatively small measurement areas that were always within one interveinal region; this resulted in higher stomatal density values compared with studies measuring stomatal density across multiple interveinal areas (e.g. Ouyang *et al.*, 2017; Chatterjee *et al.*, 2020). However, we were able to confirm that the lower stomatal densities we observed in the drought-breeding lines and released varieties was not due to larger interveinal distances in those lines; stomatal density was negatively related to interveinal distance, and the drought-breeding lines and released varieties generally showed smaller interveinal distance than IR64 (Supplementary Table S4). Nevertheless, the stomatal density values we found in all genotypes in the present study were still much higher than those reported by Caine *et al.* (2019), even when comparing only the interveinal space, indicating that this trait may still be further optimized by selection to improve rice yield under drought.

Given that this study evaluated physiological traits in some of the most recently-developed rice drought-breeding lines and released varieties available in South Asia, a key question is what traits could be further improved in this breeding pool? From the perspective of drought-escape, the typically longer time to flowering in the drought-breeding lines and released varieties compared to IR64 (Supplementary Fig. S7A) indicates potential for yield improvement by selecting for reduced time to flowering, but it would be necessary to assess the suitability of this phenotype in the respective target regions. During the development of these varieties, earlier time to flowering was specifically avoided as the breeding program aimed to develop lines with certain growth duration limits (in this case 100–120 d; Kumar *et al.*, 2014). Another trait that stands out from our study is canopy temperature under drought stress, which was negatively related to grain yield in all trials where it was measured but was not consistently lower than IR64 across genotypes (Fig. 5, Supplementary Table S8). This trait is frequently associated with a number of root traits (Henry *et al.*, 2011; Anantha *et al.*, 2016). We have previously observed a greater degree of lateral root plasticity in Sahbhagi dhan that results in lower canopy temperature in some of the environments studied here (Raipur and Cuttack; Anantha *et al.*, 2016). However, we were not able to pinpoint specific root traits related to canopy temperature or grain yield in this study (Table 3, Supplementary Fig. S6). More detailed characterization of root traits in the current drought-breeding pool is necessary. Likewise, additional traits reported to improve yield under drought in traditional varieties—especially root traits (Uga *et al.*, 2008; Hazman and Brown, 2018)—should be compared to the current drought-tolerance breeding pool in order to identify new breeding targets.

### Conclusions

This study indicates that rice breeding by direct selection for grain yield under drought stress has consistently affected flag-leaf length, stomatal density, and harvest index across varying soil moisture levels, but that the relationships between physiological traits and grain yield often depend on environmental conditions, of which atmospheric parameters stand out even more than factors affecting soil moisture. In the current breeding pool of rice genotypes characterized here, we suggest that there is potential for continued improvement through further reducing stomatal density as well as through root traits, based on the lack of consistent genotypic effects on canopy temperature under drought.

## Supplementary data

The following supplementary data are available at *JXB* online.

Table S1. List of drought-breeding lines and released varieties evaluated in this study.

Table S2. Locations of the South Asia field trials in this study.

Table S3. Environmental characteristics across the IRRI trials.

Table S4. Genotypic variation for flag-leaf length and width, and stomatal density and interveinal distance of the penultimate leaf across the South Asia trials.

Table S5. Principal component analysis across the South Asia Trials for environmental parameters.

Table S6. Principal component analysis across the South Asia Trials for each genotype and trial site.

Table S7. Correlations of grain yield with leaf traits measured in the South Asia trials.

Table S8. Genotypic variation for all traits measured across the IRRI trials.

Fig S1. Environmental characteristics across the South Asia trials.

Fig. S2. Relationships between stomatal density and grain yield as affected by environmental factors.

Fig. S3. Relationships between flag-leaf length and grain yield as affected by environmental factors.

Fig. S4. Relationships between flag-leaf width and grain yield as affected by environmental factors.

Fig. S5. Leaf traits measured across the IRRI trials.

Fig. S6. Root traits measured across the IRRI trials.

Fig. S7. Agronomic traits measured across the IRRI trials.

erab160_suppl_Supplementary_Figures_S1_S7_and_Tables_S1_S5_S7

erab160_suppl_Supplementary_Table_S6

erab160_suppl_Supplementary_Table_S8

## Data Availability

The data that support the findings of this study are openly available in Dataverse (https://dataverse.harvard.edu/dataverse/RiceResearch).
