# Supplementary material for: Rice breeding for yield under drought has selected for longer flag leaves and lower stomatal density
Source: J Exp Bot. 2021 Apr 14;72(13):4981–92. doi: 10.1093/jxb/erab160 (PMC8219034; doi:10.1093/jxb/erab160)
Supplement: erab160_suppl_Supplementary_Figures_S1_S7_and_Tables_S1_S5_S7 [file erab160_suppl_supplementary_figures_s1_s7_and_tables_s1_s5_s7.pdf]

Kumar et al
Rice drought breeding has selected for longer flag leaves and lower stomatal density
Supplemental Files

Table S1. List of drought breeding lines and released varieties evaluated in this study.

Set	Genotype	Name of released variety
Set 1	IR 93810-11-1-1-3	
	IR 93827-29-1-1-4	
	IR 93827-29-1-1-2	
	IR 74371-70-1-1	Sahbhagi dhan, BRRI dhan 56, Sukha dhan 3
	IR 95817-5-1-1-1	
	IR 93827-29-1-1-3	
	IR 93810-11-1-1-1	
	IR 82589-B-B-84-3	
	IR 93376-B-B-130	DRR dhan 44
Set 2	IR 93376-B-B-130	DRR dhan 44
	IR 97045-21-1-1-1	
	IR 82589-B-B-84-3	BRRI dhan 71
	IR 97034-21-2-1-3	
	IR 98925-11-1-2-1	
	IR 82635-B-75-2	
	IR 98976-20-1-2-1	
	IR 98976-20-1-2-2	
	IR 99739:2-1-1-2-1	
	IR 74371-70-1-1	Sahbhagi dhan, BRRI dhan 56, Sukha dhan 3
	IR 74371-54-1-1	Sukha dhan 2
	IR 93827-29-1-1-3	
Set 3	IR 108233-41-1-2-1	
	IR 108194-26-2-1-1	
	IR 108201-41-3-1-1	
	IR 93827-29-1-1-3	
	IR 93376-B-B-130	DRR dhan 44
	IR 82589-B-B-84-3	BRRI dhan 71
	IR 106298-5-1-2-2-1	
	IR 107891-B-B-1019-1-1	
	IR 108198-22-1-1-1	
	IR 108052-34-4-2-1	
	IR 74371-70-1-1	Sahbhagi dhan, BRRI dhan 56, Sukha dhan 3
	IR 106317-36-1-1-2-1	

Table S2. Locations of the South Asia field trials in this study.

Country	Site	Location of experiment	Latitude, Longitude
Bangladesh	Rajshahi	Paba, Rajshahi	24.382512 N, 88.480636 E
India	Cuttack	Cuttack, Odisha	20.4525805 N, 85.940322 E
	Hazaribag	Hazaribag, Jharkand	23.96226 N, 85.36248 E
	IIRR	Patencheru, Telangana	17.49331 N, 78.27133 E
	Patna	Patna, Bihar	25.57989 N, 85.05632 E
	Raipur	Raipur, Chhattisgarh	21.23409 N, 81.71713 E
	Sabour	Sabour, Bhagalpur, Bihar	25.225323 N 87.048402 E
	Tripura	Mirza (2016) and Khowai (2017-2018), Tripura	23.439122 N, 91.412566 E and 24.0229266 N, 91.633983611E
	Varanasi	Varanasi, Uttar Pradesh	25.255888 N, 82.986155 E
Nepal	Hardinath	Hardinath, Baniniya, Janakpurdham	26.80046 N, 85.96415 E
	Nepalgunj	Khajura, Banke	28.11412 N, 81.59347 E
	Tarahara	Tarahara, Sunsari	26.70545 N, 87.27926 E

Table S3. Environmental characteristics across IRRI trials. Values shown are the mean daily values averaged across the growing season. Rainfall values shown are during reproductive stage (60-100 days after sowing) during which the drought treatments were imposed. Soil water potential is from tensiometers installed at a soil depth of 30 cm depth. Vapor pressure deficit (VPD) was measured using the daily max. temperature and the RH values from 1400h-1500h (similar to the VPD calculation for South Asia trials).

Season	Solar rad, MJ m^{-2}	Max temp, C	Min temp, C	RH, %	VPD, kPa	Times rewatered during stress trt	Rainfall (mm) 60-100 DAS	Min soil water potential (kPa)	Ave soil water potential (kPa)
2016WS	14.03	32.75	25.10	83.34	1.200635	0	374.5	-37.5	-8.365
2017DS	14.26	30.76	23.10	88.12	1.12565	0	41.3	-72.0	-31.0
2017WS	12.04	32.43	24.47	86.05	0.872478	0	573.7	-5.0	-1.3
2018DS	15.70	31.19	23.55	82.61	2.055196	2	33.19	-36.0	-10.3
2018WS	14.50	32.67	24.72	83.10	1.375429	1	223.6	-34.25	-15.17
2019DS	17.95	30.75	18.74	85.38	1.769029	2	0	-51.25	-20.19

Table S4. Genotypic variation for flag leaf length, flag leaf width, and stomatal density and interveinal distance of the penultimate leaf across South Asia trials: lsmean values and significance groups. Set 1: 8 trials across 7 sites, Set 2: 22 trials across 12 sites, Set 3: 22 trials across 11 sites.

	**Length**		**Width**		**Stomatal density**		**Interveinal distance**	
**Genotype**	lsmean	group	lsmean	group	lsmean	group	lsmean	group
**Set 1 2016**								
IR 93810-11-1-1-1	38.4	a	2.86	a				
IR 93810-11-1-1-3	28.8	a	2.05	a				
IR 93827-29-1-1-2	33.9	a	2.17	a				
IR 93827-29-1-1-3	34	a	2.66	a				
IR 93827-29-1-1-4	34.2	a	2.55	a				
IR 95795-53-1-1-2	34.8	a	2.4	a				
IR 95817-5-1-1-1	37.9	a	2.68	a				
Sahbhagi dhan	35.1	a	2.49	a				
**Set 2 2017**								
BRRI dhan71	36.2	abc	1.41	ab	984	abc	193.728	a
IR 64	29.9	a	1.29	a	1143	c	186.688	a
IR 97034-21-2-1-3	33.1	ab	1.44	b	1025	b	180.993	ab
IR 97045-21-1-1-1	32.4	abc	1.55	b	822	a	172.379	ab
IR 97045-21-1-1-2	35	bc	1.5	b	996	ab	175.190	ab
IR 98925-11-1-2-1	38.6	c	1.3	a	1016	ab	174.595	ab
IR 98976-20-1-2-1	35.5	abc	1.35	ab	1028	abc	172.336	ab
IR 98976-20-1-2-2	33.6	ab	1.32	a	1011	ab	174.583	ab
IR 99739:2-1-1-2-1	35.7	bc	1.25	a	926	a	175.307	ab
Sahbhagi dhan	35.2	bc	1.44	b	922	a	183.071	ab
**Set 3 2018**								
BRRI dhan71	41.1	a	1.61	ab				
DRR dhan 44	32.1	a	1.31	ab				
IR 107891-B-B-1019-1-1	29.7	a	1.29	ab				
IR 108052-9-1-1-2	38.4	a	1.36	ab				
IR 108194-26-2-1-1	38.7	a	1.3	ab				
IR 108198-23-1-1-B	28.4	a	1.4	b				
IR 108198-23-24-1-B	29.9	a	1.32	ab				
IR 108201-41-3-1-1	38.3	a	1.28	ab				
IR 108231-48-3-1-1	37.6	a	1.3	ab				
IR 108233-41-1-2-1	35.6	a	1.34	ab				
IR 64	28.7	a	1.23	a				
IR 74371-70-1-1	32.3	a	1.35	ab				
IR 93827-29-1-1-2	30.5	a	1.39	b				
IR 93827-29-1-1-4	31.2	a	1.32	ab				

Table S5. Principal component analysis across South Asia Trials (Fig. 3): eigenvector values for each environmental parameter together with flag leaf length and width. Shaded values indicate the parameter with highest loading values for each principal component.

EIGENVECTORS

	PC1	PC2	PC3	PC4
**Cumulative % of variance**	30.11	48.84	62.27	71.64
**Parameter**				
% clay	0.2699	-0.0475	-0.1045	-0.0299
% sand	-0.1875	-0.1253	0.3357	-0.1593
% silt	0.1105	0.1823	-0.3782	0.2175
Available phosphorus	-0.0441	-0.2247	0.2947	0.2804
Exchangeable potassium	0.2330	-0.2465	0.0946	0.1691
pH	-0.0776	-0.0109	0.1204	0.5046
bulk density 25-30 cm	-0.1671	0.2108	0.0274	-0.2085
bulk density 5-10 cm	0.0831	-0.1256	0.3024	-0.3504
Max. penetrometer reading	-0.1185	-0.1656	0.3365	0.0429
Depth of max. penetrometer reading	0.1834	-0.0560	0.2245	0.2996
Penetrometer reading at 30 cm depth	-0.2592	-0.0800	0.0709	-0.1712
Ave. tensiometer reading 60-100 DAS	-0.1943	-0.1976	-0.3032	0.0315
Min. tensiometer reading 60-100 DAS	-0.1894	-0.1911	-0.3104	0.0602
Ave. water table depth 60-100 DAS	-0.1766	-0.2464	-0.1755	0.1507
Max. water table depth 60-100 DAS	-0.1614	-0.2679	-0.1855	0.0737
Max.daily temperature	-0.2241	0.2202	-0.0618	-0.2346
Min. daily temperature	-0.1542	0.3337	-0.0677	0.0522
Rainfall 60-100 DAS	0.1102	0.1026	0.1111	-0.0917
Relative humidity	0.1889	0.2359	0.0884	-0.0089
Vapor pressure deficit	-0.2262	-0.1587	-0.0793	-0.0475
water retention at 10 kPa	0.2812	-0.0567	-0.1785	-0.1974
water retention at 300 kPa	0.2796	-0.1758	-0.1027	-0.1391
water retention at 500 kPa	0.2725	-0.1718	-0.1149	-0.1185
water retention at 1500 kPa	0.2764	-0.2024	-0.1054	-0.1006
Leaf length	0.0660	0.2628	0.0031	0.2985
Leaf width	0.2096	0.0033	0.0756	0.0435
Grain yield	0.0882	0.3276	0.0366	0.0523

Table S6. Principal component analysis across South Asia Trials: eigenvector values for each genotype and trial site. (see attached Excel file)

Table S7. Phenotypic correlations with grain yield of the leaf traits measured in the South Asia trials (Set 1: 7 trials across 6 sites, 7 genotypes considered; Set 2: 22 trials across 12 sites, 12 genotypes considered; Set 3: 22 trials across 11 sites, 16 genotypes considered.

	**Correlations with GY**		
	**Set 1**	**Set 2**	**Set 3**
Flag leaf length	0.12	0.48***	0.29***
Flag leaf width	0.26	0.19*	0.16
Stomatal density	-	-0.18*	-

Table S8. Genotypic variation for all traits measured across IRRI trials: lsmean values and significance groups. (see attached Excel file).

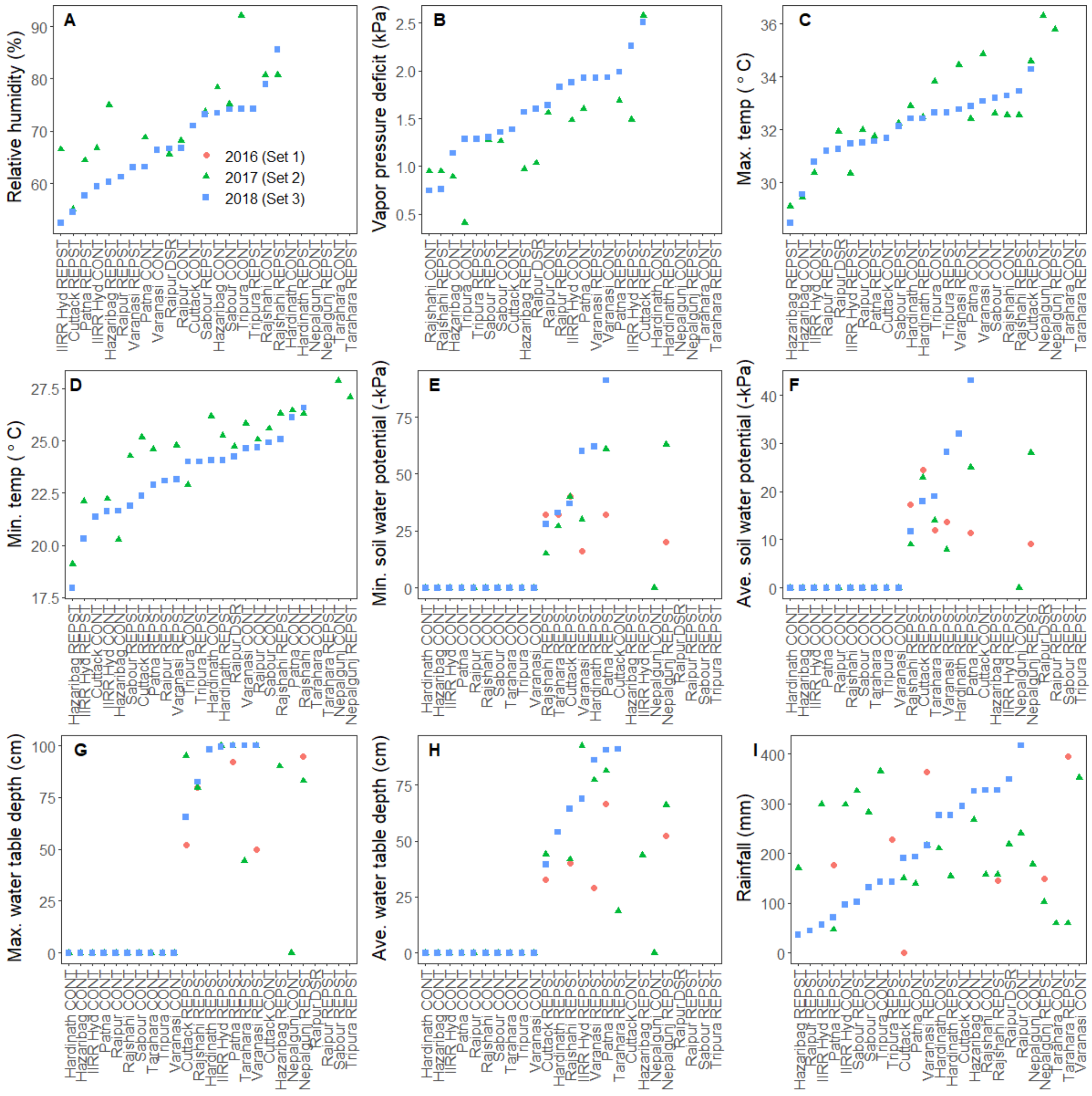

Fig S1. Environmental characteristics across South Asia trials. Rainfall values shown are during reproductive stage (60-100 days after sowing) during which the drought treatments were imposed. Soil water potential is from tensiometers installed at a soil depth of 30 cm depth. Vapor pressure deficit (VPD) was calculated from afternoon RH and max temp to estimate the maximum VPD, when possible.

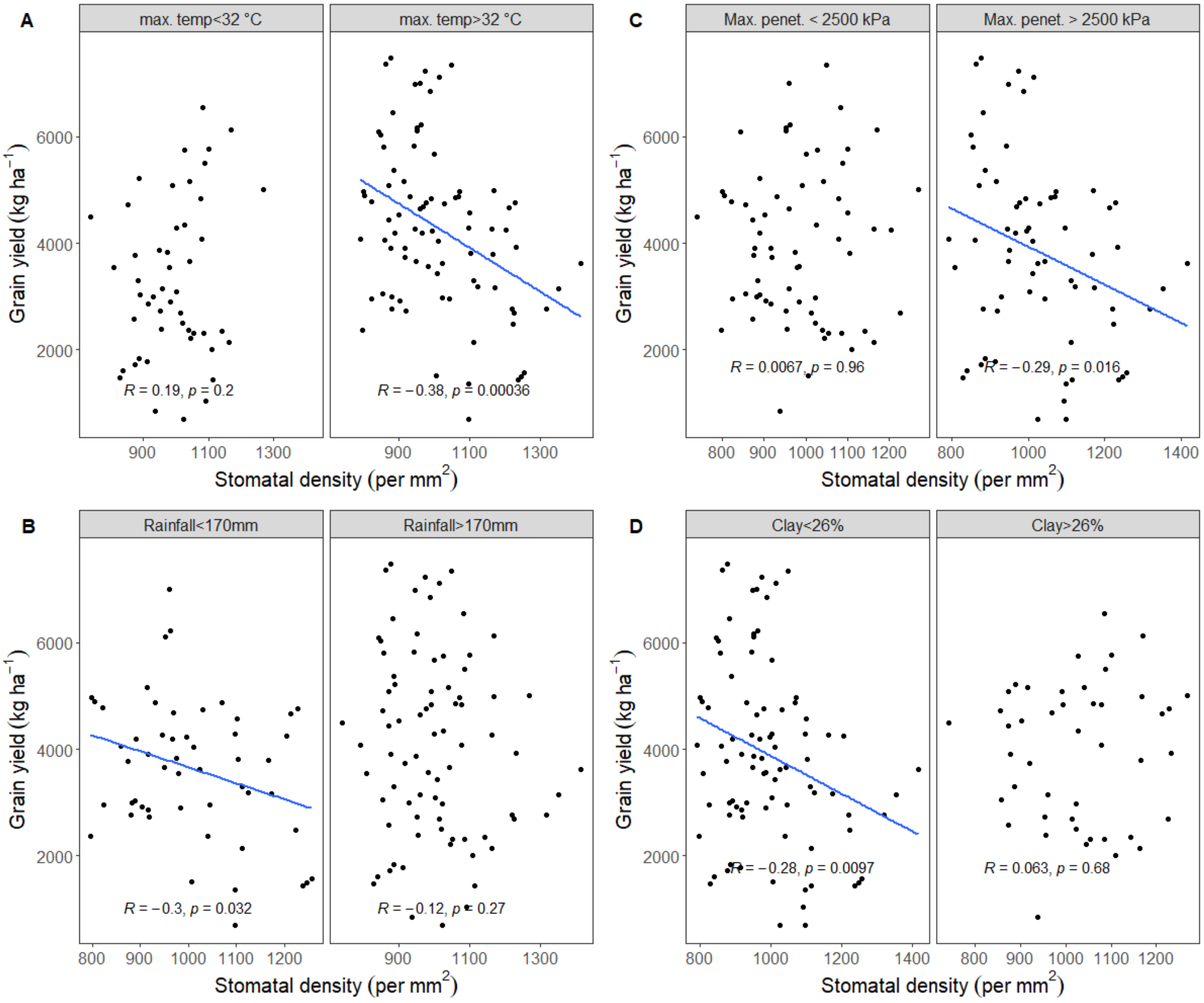

Fig, S2. The relationship between stomatal density and grain yield was affected by environmental factors: A) average max. temperature (20 trials across 11 sites) , B) rainfall (22 trials across 12 sites), C) max. soil penetrometer reading (22 trials across 12 sites), and D) soil clay content (22 trials across 12 sites). Stomatal density was measured on Set 2 (12 genotypes) in the S Asia trials, and each point represents one genotype per trial.

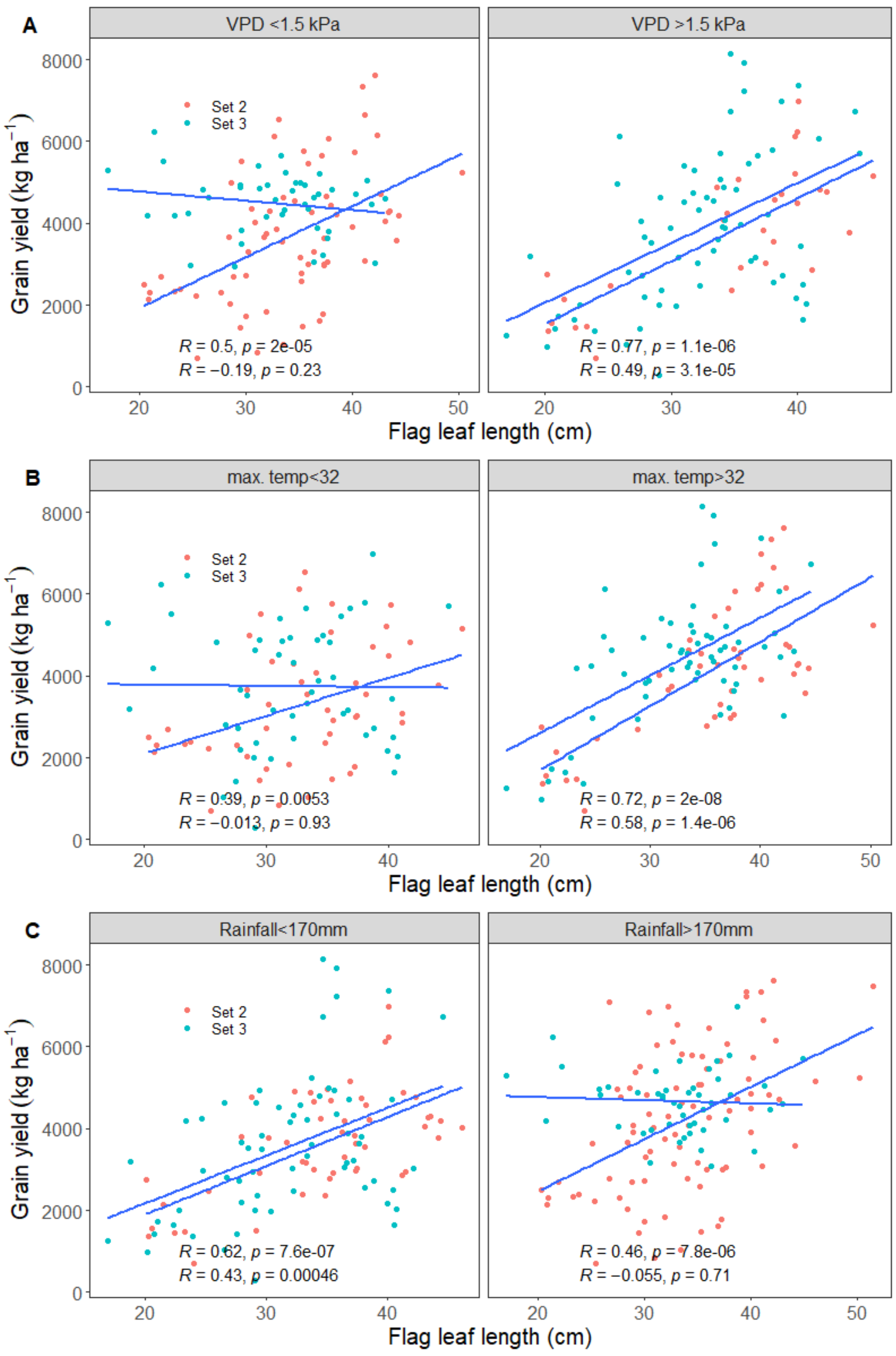

Fig, S3. The relationship between flag leaf length and grain yield was affected by environmental factors: A) VPD (Set 2: 22 trials across 12 sites, Set 3: 19 trials across 9 sites), B) average max. temperature (Set 2: 22 trials across 12 sites, Set 3: 20 trials across 10 sites), and C) rainfall (Set 2: 22 trials across 12 sites, Set 3: 19 trials across 10 sites). Genotypes are listed in Table S1, and each point represents one genotype per trial.

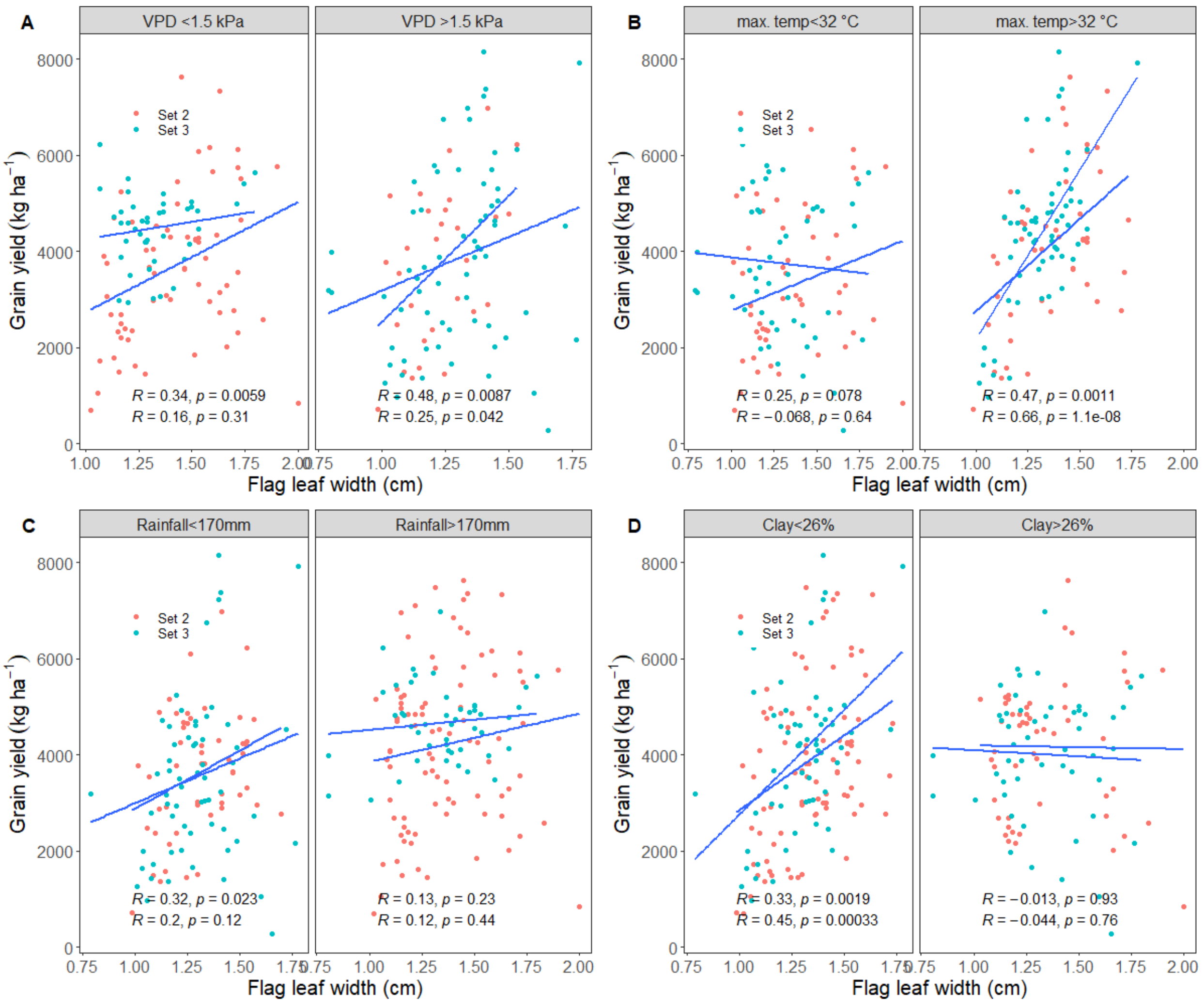

Fig, S4. The relationship between flag leaf width and grain yield was affected by environmental factors: A) VPD (Set 2: 22 trials across 12 sites, Set 3: 19 trials across 9 sites), B) average max. temperature (Set 2: 22 trials across 12 sites, Set 3: 20 trials across 10 sites), C) rainfall (Set 2: 22 trials across 12 sites, Set 3: 19 trials across 10 sites), and D) soil clay content (Set 2: 22 trials across 12 sites, Set 3: 22 trials across 11 sites). Genotypes are listed in Table S1, and each point represents one genotype per trial.

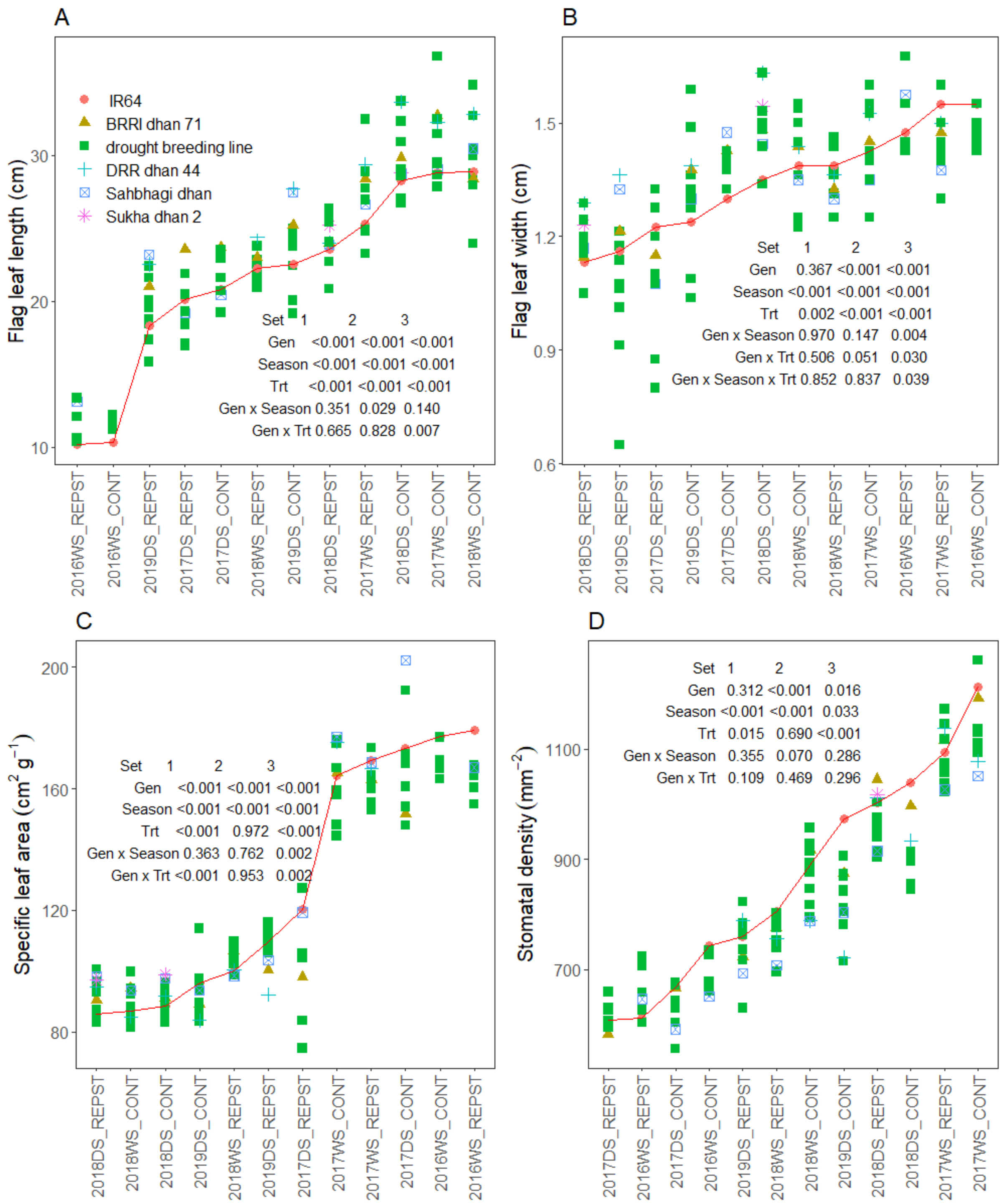

Fig. S5. Leaf traits measured across IRRI trials. A) Flag leaf length, B) Flag leaf width, C) Specific leaf area of the flag leaf, and D) stomatal density of the penultimate leaf. Set 1 was grown in 2016WS and 2017DS, Set 2 was grown in 2017WS and 2018DS, and Set 3 was grown in 2018WS and 2019DS. REPST: drought stress treatment, CONT: well-watered treatment. Genotypic differences are shown in Table S8. P-values shown are for Genotype, Site, and Treatment, and their interactions across all experiments grown for each Set.

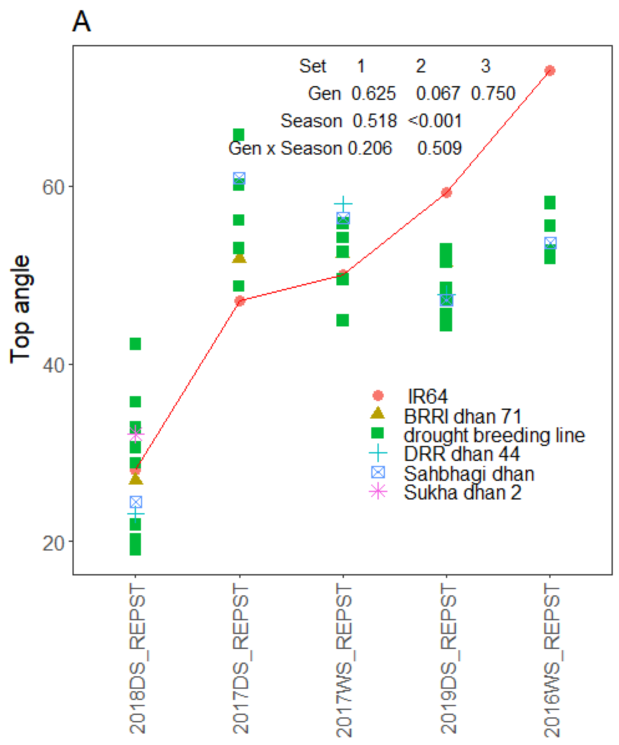

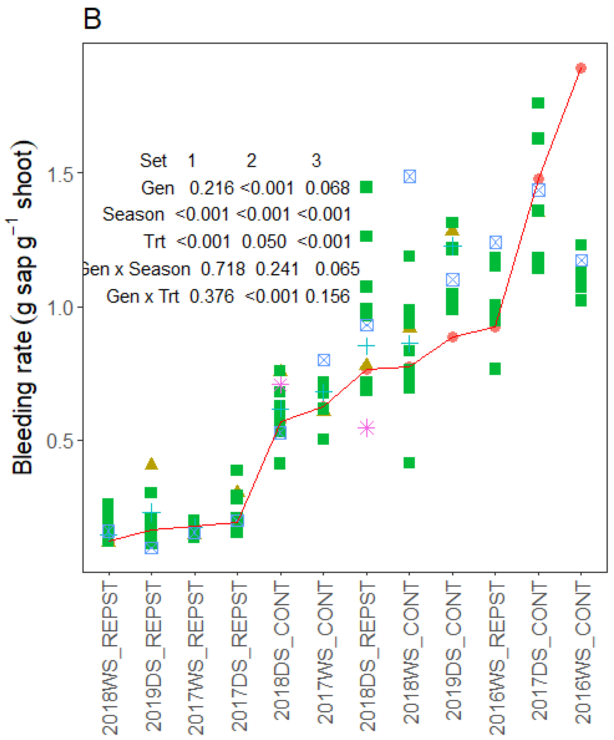

Fig. S6. Root traits measured across IRRI trials. A) root crown top angle (°), B) Sap bleeding rate. Set 1 was grown in 2016WS and 2017DS, Set 2 was grown in 2017WS and 2018DS, and Set 3 was grown in 2018WS and 2019DS. REPST: drought stress treatment, CONT: well-watered control treatment. Genotypic differences are shown in Table S8. P-values shown are for Genotype, Site, and Treatment, and their interactions across all experiments grown for each Set.

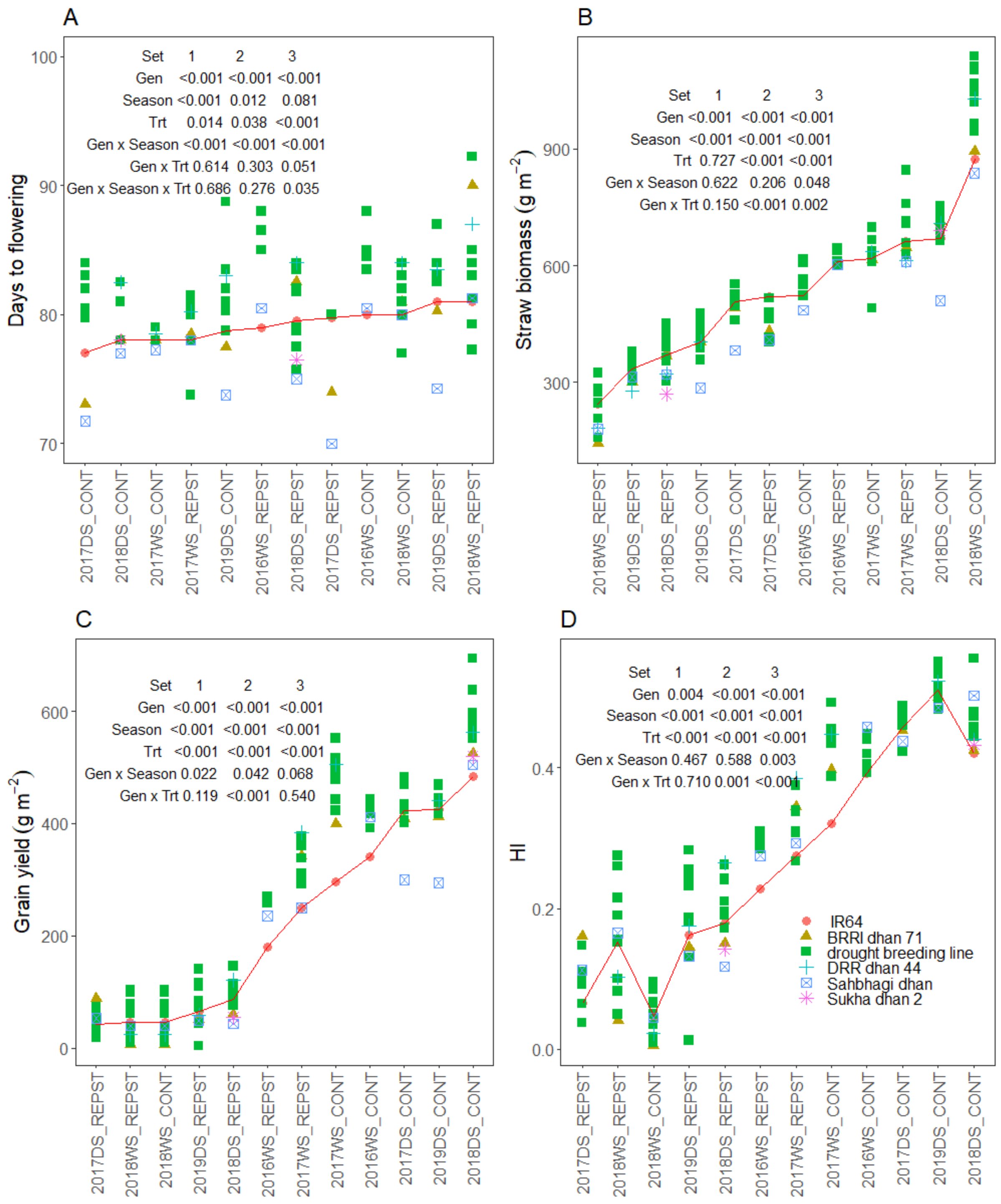

Fig. S7. Agronomic traits measured across IRRI trials. A) days to 50% flowering, B) straw biomass at harvest, C) grain yield, and D) harvest index. Set 1 was grown in 2016WS and 2017DS, Set 2 was grown in 2017WS and 2018DS, and Set 3 was grown in 2018WS and 2019DS. REPST: drought stress treatment, CONT: well-watered treatment. Genotypic differences are shown in Table S8. P-values shown are for Genotype, Site, and Treatment, and their interactions across all experiments grown for each Set.
